# *In vitro* Evaluation of Acyclovir/Chitosan Floating Systems

**DOI:** 10.3390/ma3125195

**Published:** 2010-12-06

**Authors:** Roberto Ruiz-Caro, María D. Veiga

**Affiliations:** 1 Departamento de Farmacia y Tecnología Farmacéutica, Facultad de Farmacia, Universidad Complutense de Madrid, Plaza Ramón y Cajal s/n, 28040-Madrid, Spain; E-Mail: rruizcar@farm.ucm.es (R.R.-C.); 2 Unidad de Biotransformaciones Industriales, Parque Científico de Madrid PTM, 28760-Tres Cantos, Madrid, Spain

**Keywords:** Acyclovir, chitosan, floating freeze-dried formulations, swelling behavior, controlled release

## Abstract

Chitosan (CS) floating lyophilized formulations (L) for gastric drug delivery of acyclovir (ACV) have been developed. The freeze-dried formulations were obtained from acidic aqueous suspensions prepared with different ACV/CS ratios. No changes in ACV crystallinity were observed during X-ray diffraction powder studies as a consequence of the manufacturing process. Considering that fed and fasted states modified the intragastric pH, swelling and *in vitro* dissolution studies were carried out in different acidic media (0.1 M HCl and progressive pH medium) in order to understand the influence of these physiological states on ACV/CS formulations. Swelling behavior of the floating lyophilized formulations was dependent on CS and ACV proportions within L and on medium nature due to pH dependent CS solubility. Furthermore, no interactions between ACV and CS were detected in solid state according to the X-ray studies. *In vitro* dissolution of ACV from L was influenced by the swelling behavior. However, it is feasible to optimize the ACV/CS ratios to achieve a desired formulation that releases the total quantity of ACV at a specific time. Moreover, floatability was assessed by buoyancy tests. The results demonstrated that the freeze-drying process achieved effective floating systems capable of remaining within the stomach while the total amount of ACV is released from L.

## 1. Introduction

Nowadays, the excipients choice for drug formulation is focused on natural and biocompatible products. Chitosan (CS) is a biocompatible material obtained from an abundant natural polysaccharide like chitin by deacetylation processes [1]. Several excellent reviews have compiled different methods for obtaining a wide range of chitosans with different deacetylation degrees and molecular weights which affect some of their physico-chemical parameters [2,3,4]. This polysaccharide has been used for many different applications due to the fact that it exhibits a lot of biological actions, such as hypocholesterolemic, antimicrobial and wound healing properties [4,5,6,7,8,9]. Also, in pharmaceutical technology field CS has found applicability as a potential formulation excipient acting as a disintegrant, binder or tablet coating agent. This polymer presents a swelling ability when it is placed within an aqueous media [10], and mucoadhesivity in the oral cavity [11,12,13] and in the gastrointestinal tract [14]. Moreover, it has been widely used in solid oral formulations in order to obtain sustained release systems for hydrosoluble drugs [15,16,17,18,19]. Some authors have studied its ability to improve the dissolution of poorly water-soluble drugs [20,21,22]. Furthermore, CS crosslinks with tripolyphosphate or glutaraldehyde have been used as promising excipients to achieve colonic drug delivery [23]. Finally, CS citrate has been used to develop gels for vaginal administration of poorly permeable drugs [24]. 

Human herpes simplex virus type-1 (HSV-1) infects dermal epithelial and mucosal epithelial cells, causing lesions on the epithelium of the face, generally nose or lips [25,26]. Approximately, 80% of the adult population carries HSV-1, typically asymptomatic [26,27]. Primary infection usually occurs during childhood and, subsequent to the initial outbreak, the virus enters the peripheral nervous system, where it resides permanently in a latent state of infection. Many people never see an emergence of the disease from the latent stage of infection. Nevertheless, on average, 33% of people infected with HSV-1 experience recurrences. Of these, 5% have at least one episode per month, 34% experience at least one episode every 2-11 months and 61% have at least one episode per year [26,28]. Reactivation of the virus may occur as a consequence of fever, burns, exposure to ultraviolet light, emotional or physiological stress or hormonal changes [25,26,29]. In immunocompromized individuals, outbreaks occur with increasing frequency and are harder to control [30,31,32]. Most antiherpetic treatments are composed of a nucleoside analogue that inhibits herpes virus DNA polymerase, such as acyclovir (ACV) or valacyclovir [33]. Being soluble in the gastrointestinal media, ACV is absorbed paracellularly by passive diffusion from the upper region to the duodenum or jejunum regions [34,35]. 

Some reports have suggested that the high variability of ACV effectiveness is related to saturable and dose dependent processes [36], thus its bioavailability is reduced to 15-30% [37]. In commercial available dosage forms, the amount of ACV absorbed is very low (10-20%) because of the short residence time at the absorption site. As a result, approximately 50-60 % is excreted in the feces in its unabsorbed form. Considering its short half-life (about two hours) and its incomplete absorption, ACV must be taken orally five times per day or by intravenous administration every eight hours [38]. The inclusion of absorption-enhancing excipients in formulations can enhance ACV bioavailability. ACV chemical modifications have been performed to enhance its oral bioavailability [39,40]. Luengo *et al.* [41] studied the pharmacokinetics of different preparations of ACV within β-cyclodextrins. It was found that β-cyclodextrin had no significant effect on ACV oral bioavailability as β-cyclodextrins increase the solubility of lipophilic drugs, but not the permeability of hydrophilic drugs. Attia *et al.* developed ACV niosomes to improve its oral bioavailability with positive results in rabbits [42]. 

Advantages of time-controlled release oral dosage forms in optimizing therapeutic effectiveness and reducing side effects, are their capacity to minimize the fluctuation concentrations in plasma and to reside at the site of absorption for prolonged periods of time. These properties lead to an improvement in patient compliance due to a reduction in the administration frequency and therefore a decrease of the total dose administered while maintaining similar therapeutic effects. However, conventional controlled-release dosage forms offer only limited advantages for drugs that have an absorption window in the upper small intestine (e.g., ACV [43], furosemide [44] and riboflavin [45]). Once dosage forms are evacuated from the stomach, their passage through this region is rapid, thus limiting the extent of absorption at this site. In order to enhance the absorption and bioavailability of these drugs, the residence time of the controlled-release dosage forms in the upper gastrointestinal tract needs to be prolonged [46].

Gastroretentive dosage forms, like floating systems, have achieved some success in prolonging the gastric residence time [47]. Floating dosage forms are expected to remain buoyant on gastric media because they have a lower density than gastric fluids (~1.004 g/cm^−3^). Junyaprasert and Pornsuwannapha developed hollow microspheres of ACV that were capable of floating on gastric fluids providing longer residence time at the absorption site of ACV. This is because the loaded drug starts to dissolve slowly in the stomach and is absorbed into the systemic circulation in the narrow absorption window. Thus, these forms improved ACV absolute bioavailability, resulting in a reduction of the frequency of administration, which increases patient compliance [48]. 

Moreover, another advantage of floating systems is that they do not cause problems of mucus irritation. This side effect has been reported during the development of magnetic depot tablets as an alternative to obtain ACV oral sustained release [49]. 

Whitehead *et al.* demonstrate the feasibility of a freeze-drying process by γ-scintigraphy as an effective method to develop floating systems [50]. Further, Talukder and Fassihi developed pectin freeze-dried beads and the use of confocal laser microscopy revealed hollow spaces within the beads, which allowed them to remain buoyant for over 12 hours in gastric fluid [51]. 

After considering these studies, the aim of this research is to develop floating gastric formulations of ACV/CS by freeze-drying processes, in order to obtain modulated drug release dosage forms in an attempt to increase ACV bioavailability. 

## 2. Results and Discussion

Different ACV/CS systems (L) have been prepared by a freeze-drying process. Their compositions are shown in Table 1. In order to study interactions between CS and ACV, L and raw materials employed for their preparation have been characterized in solid state by X-ray diffraction powder.

Furthermore, swelling and *in vitro* dissolution behavior of systems have been studied in order to determine their influence on ACV controlled release from L. Blank systems (B), which were prepared without ACV, have also been evaluated in swelling studies.

**Table 1:** Proportions of acyclovir (ACV) and chitosan (CS) in suspensions used to manufacture lyophilized systems.

Formulations (F)	Composition of suspensions before freeze-drying process (g/100 mL)	
	ACV	CS
L1	0.5	1
L2	0.5	2
L3	0.5	3
L4	0.5	4
L5	0.5	5
L6	2	1
L7	2	2
L8	2	3
L9	2	4
L10	2	5
B1	-	1
B2	-	2
B3	-	3
B4	-	4
B5	-	5

### 2.1. X-ray Diffraction Analysis

**Figure 1:**
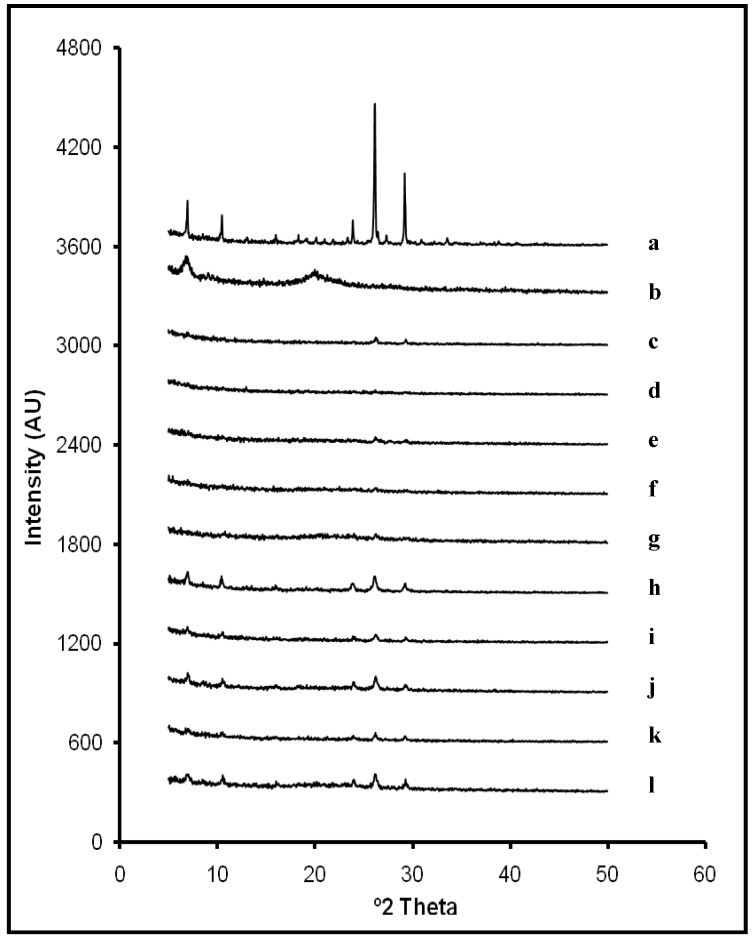
X-Ray powder diffraction patterns of ACV (**a**); CS (**b**); and systems L1 (0.5:1 w/w ACV:CS) (**c**); L2 (0.5:2 w/w ACV:CS) (**d**); L3 (0.5:3 w/w ACV:CS) (**e**); L4 (0.5:4 w/w ACV:CS) (**f**); L5 (0.5:5 w/w ACV:CS) (**g**); L6 (2:1 w/w ACV:CS) (**h**); L7 (2:2 w/w ACV:CS) (**i**); L8 (2:3 w/w ACV:CS) (**j**); L9 (2:4 w/w ACV:CS) (**k**); and L10 (2:5 w/w ACV:CS) (**l**); in solid state.

Figure 1 depicts X-ray diffraction powder patterns of raw ACV and CS and lyophilized systems in solid state. The diffraction pattern of ACV revealed its characteristic peaks at 7.0°, 10.5°, 23.9°, 26.2° and 29.2° 2θ. As expected, CS did not exhibit any diffraction peak because of its amorphous nature. All ACV/CS freeze-dried formulations reflected similar diffraction patterns, where ACV characteristic peaks were detected with less intensity. This indicates that the freeze-drying process did not modify the crystalline structure of ACV, although peaks’ intensity was clearly affected which could be due to crystallite size. Diffraction patterns of the formulations from L1 to L5 showed less intensity in ACV characteristic peaks due to the lower proportion of ACV compared to the X-ray diffraction patterns of systems with a higher amount of ACV (L6 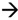 L10). Furthermore, slight displacements on ACV characteristic peaks were detected on some diffraction patterns (from 26.2° to 26.3° 2θ; from 29.2° to 29.3° 2θ and from 23.9° to 24.0° 2θ). It can be related to some modifications in the crystallite size during the lyophilization process. However, results suggest that there are no interactions between ACV and CS within systems in the studied ratios.

### 2.2. Swelling Test

**Figure 2:**
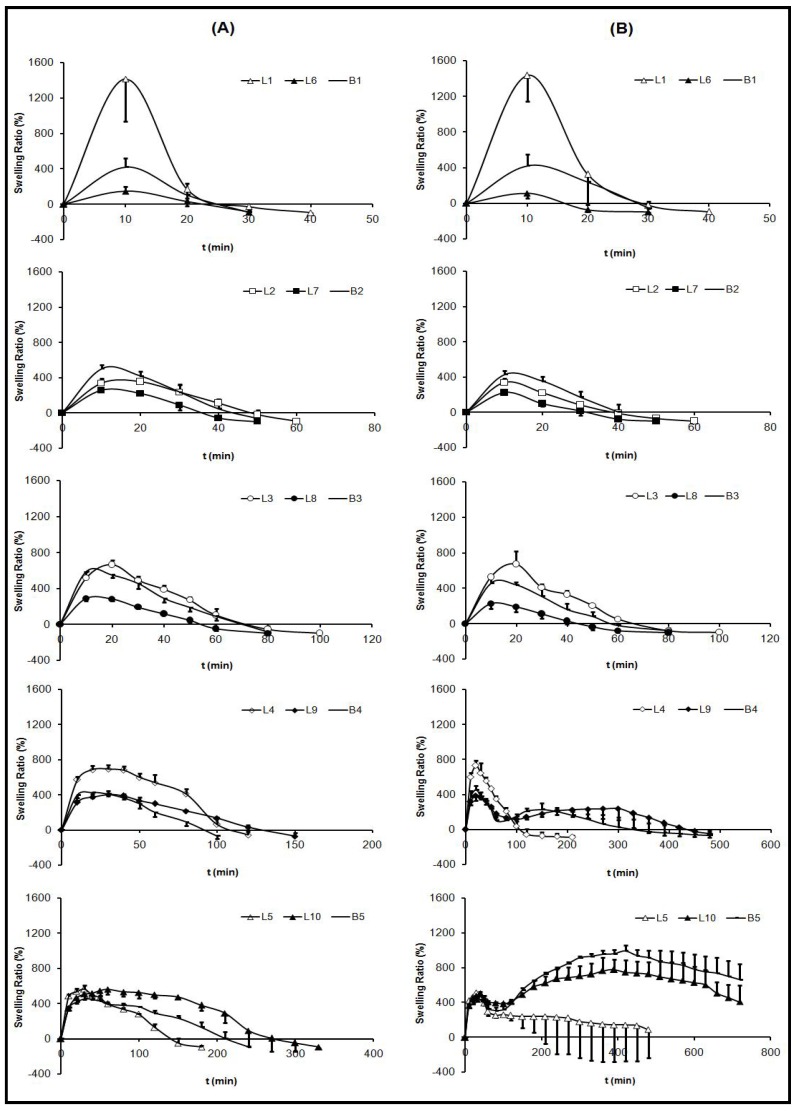
Swelling ratio of freeze-dried systems B1 (1% CS), B2 (2% CS), B3 (3% CS), B4 (4% CS), B5 (5% CS), L1 (0.5:1 w/w ACV:CS), L2 (0.5:2 w/w ACV:CS), L3 (0.5:3 w/w ACV:CS), L4 (0.5:4 w/w ACV:CS), L5 (0.5:5 w/w ACV:CS), L6 (2:1 w/w ACV:CS), L7 (2:2 w/w ACV:CS), L8 (2:3 w/w ACV:CS), L9 (2:4 w/w ACV:CS) and L10 (2:5 w/w ACV:CS) in 0.1 M HCl medium (pH 1) (**A**) and in progressive pH medium (pH 1.5 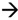 pH 4) (**B**).

Figure 2 shows the swelling ratios of systems in HCl medium (pH 1) (**A**) and in progressive pH medium (**B**), where each positive swelling ratio value indicates that, at this time, the swollen system weight was higher than the dry system weight (t = 0). On the other hand, each negative swelling ratio value indicated that the weight of the swollen system was lower than the weight of the dry system (t = 0). When t = 0, the swelling ratio value is 0 in all systems, due to the application of the equation shown in the experimental section. 

The same two phase swelling behavior was observed in all the systems. The first phase of imbibition corresponds to a process of weighing gain, followed by a second phase of CS dissolution, resulting in a reduction of weight. *In vitro* swelling behavior has demonstrated to be influenced by the ACV/CS ratios and medium nature. The maximum swelling ratios of every system and the time required for obtaining these values in both media are included in Table 2. 

**Figure 3:**
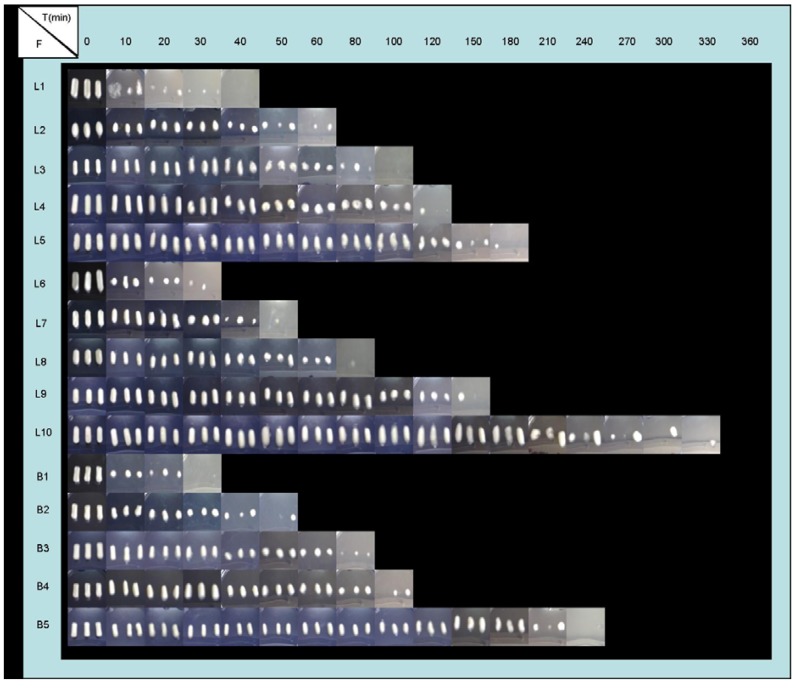
Evolution of freeze-dried formulations (F) B1 (1% CS), B2 (2% CS), B3 (3% CS), B4 (4% CS), B5 (5% CS), L1 (0.5:1 w/w ACV:CS), L2 (0.5:2 w/w ACV:CS), L3 (0.5:3 w/w ACV:CS), L4 (0.5:4 w/w ACV:CS), L5 (0.5:5 w/w ACV:CS), L6 (2:1 w/w ACV:CS), L7 (2:2 w/w ACV:CS), L8 (2:3 w/w ACV:CS), L9 (2:4 w/w ACV:CS) and L10 (2:5 w/w ACV:CS) for swelling study in 0.1 M HCl medium (pH 1).

**Figure 4:**
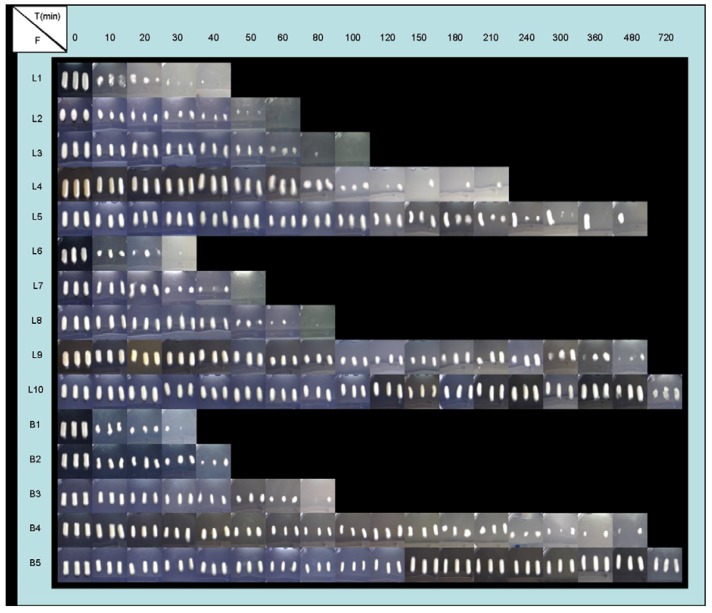
Evolution of freeze-dried formulations (F) B1 (1% CS), B2 (2% CS), B3 (3% CS), B4 (4% CS), B5 (5% CS), L1 (0.5:1 w/w ACV:CS), L2 (0.5:2 w/w ACV:CS), L3 (0.5:3 w/w ACV:CS), L4 (0.5:4 w/w ACV:CS), L5 (0.5:5 w/w ACV:CS), L6 (2:1 w/w ACV:CS), L7 (2:2 w/w ACV:CS), L8 (2:3 w/w ACV:CS), L9 (2:4 w/w ACV:CS) and L10 (2:5 w/w ACV:CS) for swelling study, in progressive pH medium (pH 1.5 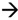 pH 4).

Influence of CS proportion: By studying blank systems (B), the characteristic CS swelling behavior has been determined. When increasing CS proportion within the system, lower swelling speed was obtained, lower amount of medium is imbibed, and therefore, a lower dissolution rate of CS was detected. Systems with lower proportions of polymer (B1, B2 and B3) showed clear differences between imbibition and dissolution phases. While, systems with high CS proportions (B4 and B5) displayed overlapped phases, because these systems were more compact and, as a consequence, they were able to maintain their shape for a prolonged time (Figure 3 and Figure 4). 

**Table 2:** Maximum swelling ratios (SR) and the time required for obtaining the values of freeze-dried systems: B1 (1% CS), B2 (2% CS), B3 (3% CS), B4 (4% CS), B5 (5% CS), L1 (0.5:1 w/w ACV:CS), L2 (0.5:2 w/w ACV:CS), L3 (0.5:3 w/w ACV:CS), L4 (0.5:4 w/w ACV:CS), L5 (0.5:5 w/w ACV:CS), L6 (2:1 w/w ACV:CS), L7 (2:2 w/w ACV:CS), L8 (2:3 w/w ACV:CS), L9 (2:4 w/w ACV:CS) and L10 (2:5 w/w ACV:CS), in 0.1 M HCl medium (pH 1) and in progressive pH medium (pH 1.5 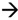 pH 4).

Formulations	0.1 M HCl	medium	progressive pH	medium
	Maximum SR (%)	T max (min)	Maximum SR (%)	T max (min)
B1	419.16 ± 99.16	10	420.41 ± 125.25	10
B2	501.50 ± 47.11	10	428.44 ± 45.24	10
B3	587.41 ± 52.87	10	457.51 ± 75.75	10
B4	425.75 ± 61.66	20	453.11 ± 43.88	20
B5	454.09 ± 43.44	30	992.05 ± 63.13	420
L1	1413.92 ± 476.74	10	1433.65 ± 289.10	10
L2	337.03 ± 52.79	10	338.01 ± 48.10	10
L3	666.92 ± 50.33	20	673.52 ± 146.14	20
L4	694.25 ± 41.99	30	730.73 ± 58.05	20
L5	565.66 ± 41.08	30	511.96 ± 63.49	30
L6	146.33 ± 54.28	10	111.92 ± 58.52	10
L7	258.19 ± 11.41	10	225.02 ± 24.29	10
L8	287.95 ± 24.75	10	227.24 ± 59.78	10
L9	404.99 ± 37.59	30	387.88 ± 59.51	10
L10	560.52 ± 58.53	60	777.00 ± 114.73	390

Nevertheless, in progressive pH medium, four different phases can be observed: An imbibition process in pH 1.5 medium; a CS dissolution process in pH 1.5; a new imbibition process when achieving pH 4 and finally; a slow CS dissolution in pH 4 medium which demonstrated to last longer than 12 hours. Due to a rise in the pH of the medium, the CS dissolution was stopped and again systems absorbed medium. The aspect of the systems during swelling tests (Figure 3 and Figure 4) was in accordance with the results obtained from swelling ratio (Figure 2).

Influence of medium nature: Due to the pH-dependent CS solubility [1,4], some influence of medium nature was expected on swelling behavior. Unexpectedly however, systems with lower proportions of CS (1-3%) exhibited similar swelling behavior in both media, due to completing imbibition and dissolution phases within one hour before progressive pH medium was reached. Instead, formulations with higher proportions of CS (B4, L4, L9, B5, L5 and L10) exhibited a prolonged swelling process (imbibitions phase), and in these cases, when increasing the pH medium (progressive pH medium), differences among swelling ratios were observed. The imbibition and CS dissolution phases could be clearly differed and occurred for less than six hours in HCl medium (pH 1).

Influence of ACV proportion: The incorporation of ACV within the systems modified the swelling behavior of CS due to ACV crystallites altering the linear structure of CS chains and, as a consequence, their ability to absorb aqueous media. A correlation between ACV/CS swelling behavior ratios can be defined. 0.5% ACV systems achieved higher weights in the imbibition phase than 2% ACV systems due to their capacity of water absorption. Regarding the CS dissolution phase, systems with low CS proportions (L1, L2, L3, L6, L7 and L8) showed similar behaviors, although in systems with 0.5 % ACV (L1, L2 and L3), the required time compared to the total CS dissolution is slightly higher, as can be seen in Figure 3 and Figure 4. On the other hand, ACV systems with high amounts of CS (L4, L9, L5 and L10) exhibited a delay in the CS dissolution process, and also it was more prolonged in 2% ACV systems (L9 and L10). The system that required longer to be dissolved was L10, which, even after 6 hours, remained undissolved.

### 2.3. Buoyancy Test

All the studied lyophilized systems floated immediately upon contact with the release medium, showing no lag times in buoyancy of the systems. The low density of the systems could be a by-product of the freeze-drying process. 

### 2.4. Dissolution Test

**Figure 5:**
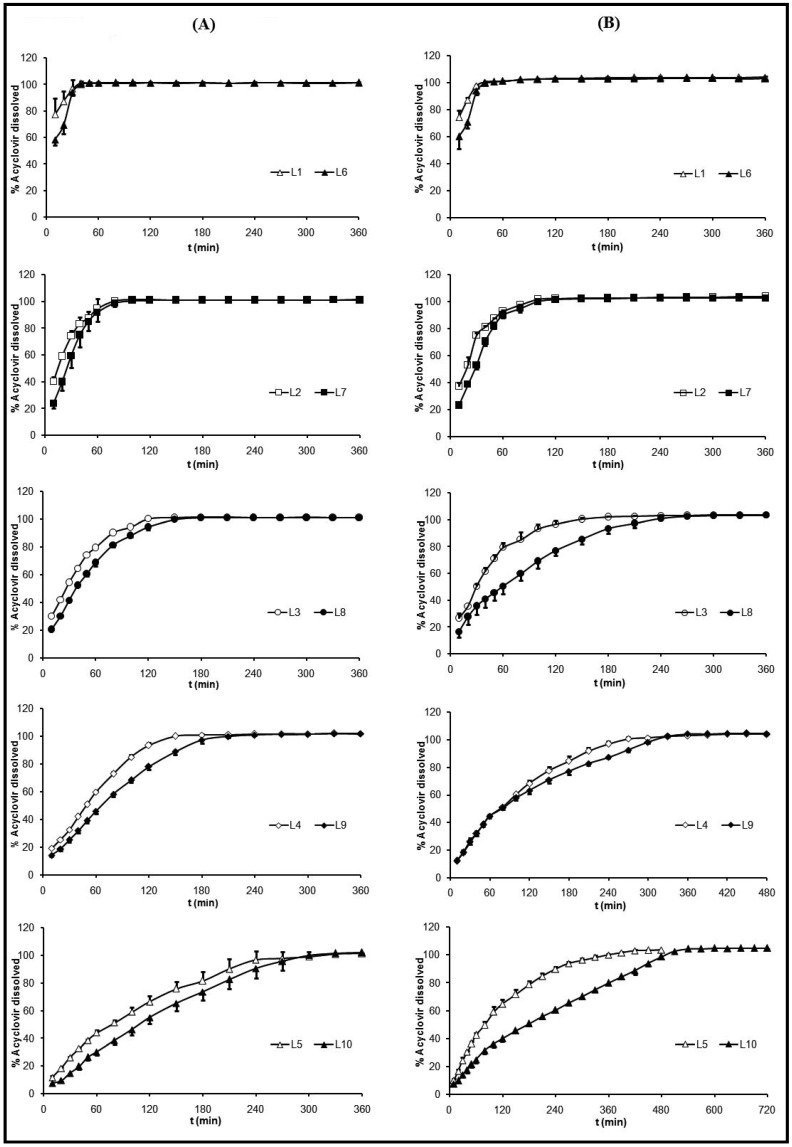
Dissolution profiles of freeze-dried systems (L) L1 (0.5:1 w/w ACV:CS), L2 (0.5:2 w/w ACV:CS), L3 (0.5:3 w/w ACV:CS), L4 (0.5:4 w/w ACV:CS), L5 (0.5:5 w/w ACV:CS), L6 (2:1 w/w ACV:CS), L7 (2:2 w/w ACV:CS), L8 (2:3 w/w ACV:CS), L9 (2:4 w/w ACV:CS) and L10 (2:5 w/w ACV:CS) in 0.1 M HCl medium (pH 1) (**A**) and in progressive pH medium (pH 1.5 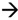 pH 4) (**B**).

Release profiles of ACV from freeze-dried formulations in HCl medium (pH 1) are represented in Figure 5 (**A**) and in progressive pH medium in Figure 5 (**B**). ACV release profiles from systems were influenced by the same factors as swelling behavior: CS and ACV ratios and medium nature. 

Influence of CS proportion: ACV release profile was clearly influenced by CS proportion, as can be observed in Figure 5. As in the swelling phases, CS imbibition was the first step in the dissolution process. For this reason, an increase in the CS proportion produced more compact swollen systems and, therefore, more sustained profiles were obtained due to more time being required for dissolution and diffusion of ACV molecules within the system. On one hand, in HCl medium, at 40 minutes, the system L1 (low CS ratio) exhibited a dissolution profile where 100 % of ACV was dissolved. While system L3 (medium CS ratio) required 120 minutes and system L5 (high CS ratio) required 330 minutes (Figure 5(**A**)). 

On the other hand, in progressive pH medium, the influence of CS proportions in drug released was also detected (Figure 5(**B**)). The higher the CS ratio within the systems, the more sustained the releasing ACV profile. A conventional release profile was obtained from systems L1, L2, L6 and L7, while a sustained release profile was obtained from systems L4, L5, L9 and L10.

Influence of ACV proportion: Dissolution profiles obtained in HCl medium (pH 1) from systems with the same amount of CS and different concentration of ACV (L1 and L6, L2 and L7, L3 and L8, L4 and L9 and L5 and L10) are similar, because ACV is soluble in this medium. Furthermore, the total drug dissolution from every system was only obtained when the freeze-dried formulations had completely lost their initial shape and aspect (Figure 3). However, at a progressive pH medium (pH 1.5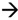 4), the influence of ACV proportion over its selfsame dissolution was a little more remarkable. At 120 minutes the percentages of released ACV from the systems with an equal amount of CS and different amounts of ACV were: from L3, 96.7% and from L8, 76.8%; from L4, 68.1 % and from L9, 63.1%; from L5, 64.6% and from L10, 40.0%. These different behaviors were due to the pH dependent ACV dissolution [52]. 

Influence of medium nature: The influence of dissolution medium composition in ACV release mechanism is clear in the systems with high proportions of CS and ACV, where the required time to obtain total drug dissolution was from 300 to 480 minutes (L5) and from 360 to 540 minutes (L10). Due to the fact that CS exhibits pH-dependency on its solubility [1,4] and on imbibition phase, which was previously discussed in the swelling behavior section, ACV dissolution rate from the systems has shown to be influenced by the medium. Therefore, all the systems in HCl medium (pH 1) exhibited total drug dissolution in less than six hours, while in progressive pH medium the duration of dissolution tests was prolonged to 12 hours in order to achieve complete drug dissolution of all formulations. During the first hour, similar dissolution rates were obtained in both media, due to the similarity of the pH values. However, after pH was changed from 1.5 to 4, in the dissolution tests with progressive pH medium, the systems exhibited an ACV release more prolonged, being even more controlled in the systems with high amounts of ACV and CS. This could be related to the durability of the formulation in the progressive pH medium as previously explained in the four steps in the swelling behavior section. 

## 3. Experimental Section

### 3.1. Materials

Acyclovir (batch No. 14070620002) was a generous gift from CINFA (Pamplona, Spain). Chitosan (batch No. 8826900003) with a viscosity of 92.0 cSt and a deacetylation degree of 97.0% was purchased from Guinama (Valencia, Spain). All other reagents were of analytical grade. PVC casts were a generous gift from Roche (Leganés, Madrid, Spain). 

### 3.2. Preparation of ACV/CS Freeze-Dried Formulations (L)

ACV, previously sieved through a mesh size of 100 μm, was dispersed into an aqueous solution of acetic acid (1% v/v). Then, CS (previously sieved through a mesh size of 100 μm) was added to ACV suspension. The system was gently mixed with a magnetic stirrer until a homogeneous suspension was obtained. To prepare freeze-dried dosage forms, different suspensions of ACV/CS were dispensed into PVC casts and afterwards they were freeze-dried (Lio-Labor^®^; Telstar, Barcelona, Spain) for 48 hours reaching a freezing temperature, a sublimation pressure and a sublimation temperature into a chamber of −45 °C, 4.54 10^−4^ atm and from −45 to 25 °C, respectively. Blank freeze dried formulations (B1 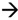 B5) were also prepared, as described above, in order to compare them to the ACV/CS formulations in the characterization studies. The dimensions of systems were measured, and the results were 22.5 ± 0.7 mm (length), 8.9 ± 0.3 mm (width) and 6.1 ± 0.2 mm (thickness) (n = 15). 

### 3.3. Characterization of ACV/CS Lyophilized Formulations 

#### 3.3.1. X-ray diffraction analysis

Powder X-ray diffraction patterns of raw materials and ACV/CS freeze-dried formulations were recorded by using an automated Philips^®^ X’Pert X-ray diffractometer (Almelo, Netherlands). Samples were irradiated with monochromatized Cu-Kα radiation and analyzed between 2*θ* angles of 5 and 40°. The current, the voltage and the time per step, were 55 mA, 40 mV and 1 s, respectively. A software package attached with the diffractometer was used to calculate the peak heights of all diffraction patterns (CAI, DRX, UCM). Powder X-ray diffraction patterns were measured in order to evaluate the crystalline/amorphous character of pure ingredients untreated and ACV/CS freeze-dried formulations.

#### 3.3.2. Swelling test

Swelling ratio was evaluated as weight gain at a medium temperature of 37 ± 0.1 °C. As the gastric pH in fasted subjects is 1.1 ± 0.15 [53,54,55] and in fed state is 2.0 to 6.0 [56], the swelling, the buoyancy and the dissolution experiments were carried out in 0.1 M HCl (pH 1) and in progressive pH medium (1.5 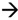 4). This progressive medium was composed of an aqueous mixture of 0.05 M hydrochloric acid 37%, 0.05 M ortho-phosphoric acid 85% and 0.05 M acetic acid glacial with a final pH value of 1.5, which was maintained during the first hour. After this hour, a sufficient quantity of 10 M NaOH was added until pH reached a value of 4.0, which was maintained until the test was finished. 

The maximum duration of the tests was six hours in 0.1 M HCl medium (pH 1) and 12 hours in progressive pH medium. At specific time intervals, the samples were removed from test medium and were blotted with filter paper to absorb the excess of liquid on sample surface. At the same time, photographs were taken with a digital camera (Fujifilm^®^ Finepix A345 (Tokyo, Japan) 4.1 Megapixels) to observe the appearance and evolution of all formulations in contact with the media. The swelling ratio (SR%) of every sample was calculated according to the equation [57]:

SR% = [(Ls-Ld)/Ld] x 100%
(1)

where Ls and Ld were the weights of the swollen and dried samples respectively. All swelling tests were performed in triplicate.

#### 3.3.3. Buoyancy test

All systems prepared were observed in order to determine their buoyancy ability, using the paddle method at a rotation speed of 100 rpm with a Sotax^®^ AT-7 dissolution apparatus (Basel, Switzerland) in 900 mL of either 0.1 M HCl (pH 1) or progressive pH medium at 37 °C. 

The maximum duration of assays was six hours in 0.1 M HCl medium (pH 1) and 12 hours in progressive pH medium. In every case, the samples performed contained 20 mg of ACV. The position or location of the lyophilized systems during buoyancy tests was checked visually every hour. All buoyancy tests were performed in triplicate.

#### 3.3.4. Dissolution test

A Sotax^®^ AT-7 dissolution apparatus (Basel, Switzerland) with paddles was employed to carry out all tests. The paddle speed, volume of the dissolution medium and experimental temperature were 100 rpm, 900 mL of either 0.1 M HCl (pH 1) or progressive pH medium and 37 ± 0.1 °C, respectively. A sample quantity of 20 mg of ACV (previously sieved, size < 100 μm), or its equivalent amount from lyophilized systems, were used for all dissolution tests. The maximum duration of the tests was six hours in 0.1 M HCl medium (pH 1) and 12 hours in progressive pH medium. Samples were withdrawn at specific time intervals and filtered using Whatman^®^ filter paper (type 42). The quantity of dissolved ACV was determined by UV spectroscopy at a wavelength of 255 nm (pH 1 and pH 1.5) and at 251.5 nm (pH 4), using in all cases a Beckman^®^ DU-7 spectrophotometer (Brea, CA, U.S.). Three replicates of each dissolution test were carried out. The fact that there was no change in the λ_max_ of ACV because of the presence of CS had previously been checked.

## 4. Conclusions

The freeze-drying process enables ACV/CS floating systems to remain in gastric medium (fasted and fed states) until the total amount of ACV is released from the system. The amount of CS incorporated in the systems condition the swelling behavior and, consequently, the ACV release profile. These systems are capable of fast release formulations, releasing 100% of ACV within one hour (L1, L2, L6 and L7), or a more controlled release formulation which can release ACV over three hours or longer (L4, L5, L9 and L10). 
